# Assessment value of blood pressure variability combined with serum tau protein for the prognosis of patients with traumatic brain injury

**DOI:** 10.3389/fneur.2026.1790987

**Published:** 2026-05-04

**Authors:** Feng Lin, Chao Luo, Yikuan Gao

**Affiliations:** 1 Department of Neurosurgery, Yongzhou Central Hospital Lengshuitan Campus, Yongzhou, Hunan, China; 2 Department of Emergency, Yongzhou Central Hospital Lengshuitan Campus, Yongzhou, Hunan, China

**Keywords:** blood pressure variability, disease severity, prognosis, tau protein, traumatic brain injury

## Abstract

**Objective:**

To investigate the prognostic value of blood pressure variability (BPV) combined with serum tau protein in patients with traumatic brain injury (TBI).

**Methods:**

A total of 205 TBI patients were prospectively enrolled and categorized into mild, moderate, and severe groups based on their Glasgow Coma Scale (GCS) scores at admission. BPV [coefficient of variation for systolic and diastolic blood pressure (CV-SBP, CV-DBP)] and serum tau protein levels were measured. Their correlations with disease severity and 28-day prognosis were analyzed. Univariate and multivariable logistic regression analyses identified prognostic factors, and receiver operating characteristic (ROC) curves evaluated the predictive performance of individual indicators and their combination.

**Results:**

Levels of CV-SBP, CV-DBP, and tau protein significantly increased with TBI severity (all *p* < 0.017) and showed positive correlations with the degree of injury (all *p* < 0.001). Multivariate analysis identified pupillary dilation, midline shift ≥5 mm, elevated CV-SBP, elevated CV-DBP, and elevated tau protein as independent risk factors for poor prognosis. The areas under the ROC curve (AUC) for predicting poor prognosis were 0.778 for CV-SBP, 0.765 for CV-DBP, 0.799 for tau protein, and 0.921 for the combination model. The combined model’s predictive efficacy was significantly superior to any single indicator alone (all *p* < 0.05).

**Conclusion:**

Both BPV and serum tau protein are reliable predictors of severity and poor prognosis in TBI patients. Their combination demonstrates superior incremental predictive performance compared with any single indicator in this cohort, but requires external validation before clinical implementation.

## 1 Introduction

Traumatic brain injury (TBI) is a major global cause of mortality and disability among young adults, imposing a substantial social and economic burden (1). Despite significant advances in neurocritical care, surgical techniques, and comprehensive management protocols, the prognosis for TBI patients remains concerning, particularly for those with severe TBI, where mortality rates persist at high levels, and survivors often experience serious neurological deficits (2, 3). This considerable uncertainty in predicting outcomes presents a severe challenge for both clinicians and patients’ families. Consequently, there is a critical need to develop and apply tools that can early, objectively, and accurately assess the severity and predict the clinical outcome of TBI. Such tools are paramount for risk stratification, guiding individualized precision treatment, optimizing the allocation of medical resources, and ultimately improving patients’ long-term quality of life (4). While the Glasgow Coma Scale (GCS) and conventional imaging provide essential baseline information, their sensitivity and specificity for predicting prognosis are insufficient, underscoring the urgent need to identify novel indicators that dynamically reflect the complex pathophysiology of TBI.

Researchers have long sought predictive factors for TBI prognosis across different dimensions. Regarding physiological parameters, blood pressure management is a cornerstone of TBI care. However, traditional strategies have primarily focused on maintaining specific absolute blood pressure targets, often overlooking the dynamic fluctuations of blood pressure over time. Blood pressure variability (BPV), a metric that quantifies the degree of blood pressure fluctuation, has gained increasing attention in the field of cerebrovascular diseases in recent years. Substantial evidence confirms that increased BPV is independently associated with poor outcomes in patients with acute ischemic stroke and spontaneous intracerebral hemorrhage (5, 6). The underlying mechanism involves BPV’s sensitivity in reflecting impairment of cerebral autoregulation (CA). Significant blood pressure fluctuations can lead to instability of cerebral perfusion pressure, thereby exacerbating cerebral ischemia, reperfusion injury, and blood–brain barrier (BBB) disruption (7, 8). This concept is gradually being recognized in the field of TBI. For instance, Li et al. (9) found that systolic BPV (CV ~ SBP~) was associated with the progression of intraparenchymal hematoma in TBI patients, and Zhang et al. (10), using a large database analysis, also suggested a predictive value of BPV for TBI outcome. Nevertheless, existing studies are largely retrospective or focus on specific complications. More high-level evidence is required to clarify the dynamic changes of BPV across TBI severities and its value as an independent predictor in prospective cohorts.

In the realm of biomarkers, advances in molecular biology have fueled the search for blood-based markers that specifically reflect neuronal injury. Among these, serum tau protein shows considerable promise. Tau is a microtubule-associated protein predominantly located in neuronal axons, crucial for maintaining cytoskeletal stability and axonal transport. When TBI causes axonal injury, tau protein is released into the extracellular space and subsequently enters the bloodstream, making its serum concentration a sensitive indicator of neuronal damage, particularly diffuse axonal injury (11, 12). Multiple studies have demonstrated that serum tau protein levels are significantly elevated in TBI patients and correlate with injury severity. For example, Whitehouse et al. (13) reported an association between serum tau protein and self-reported outcomes at 6 months in patients with mild TBI, and other studies have linked it to mortality in severe TBI patients (14). Although these findings are encouraging, most studies have treated tau protein as an isolated indicator, failing to fully elucidate its intrinsic relationship and synergistic effect with functional parameters reflecting systemic physiological status, such as BPV. The pathophysiology of TBI is a multidimensional and multi-level entity, involving both microscopic molecular and cellular damage and macroscopic physiological dysfunction. Combining a “static” biomarker of neuronal injury with a “dynamic” physiological parameter of cardiovascular regulation to construct a multidimensional assessment system holds the potential to more comprehensively capture the pathophysiological essence of TBI, thereby enabling more accurate prognosis prediction (15).

Based on the aforementioned research gap, this study innovatively proposes the combined detection of BPV and serum tau protein to explore their synergistic value in assessing the prognosis of TBI patients. We hypothesize that the state of cerebral perfusion instability represented by BPV and the degree of neuronal axonal injury represented by serum tau protein mutually reinforce each other following TBI, collectively determining the patient’s final outcome. To this end, we conducted a prospective observational study. We systematically enrolled TBI patients of varying severities, collected detailed baseline clinical data, precisely calculated BPV indices (CV ~ SBP~ and CV ~ DBP~) via 24-h ambulatory blood pressure monitoring, and measured serum tau protein levels at admission using enzyme-linked immunosorbent assay (ELISA). Subsequently, we analyzed the correlation of these two indicators with TBI severity and identified independent risk factors affecting the 28-day prognosis through univariate and multivariable logistic regression analyses. Finally, we utilized receiver operating characteristic (ROC) curves to compare the predictive efficacy of BPV, serum tau protein, and their combined model for poor prognosis, aiming to validate whether their combined application provides superior predictive value compared to any single indicator, thereby offering a novel and comprehensive reference model for the clinical prognostic assessment of TBI.

## 2 Materials and methods

### 2.1 Clinical data

Clinical data were collected prospectively/retrospectively from the electronic medical record system by trained investigators using a standardized case report form. Collected variables included demographics (age, sex), injury-related characteristics, comorbidities, neurological examination findings at admission (including pupillary response), neuroimaging findings (e.g., midline shift and other CT indicators as available), laboratory indicators at admission, and in-hospital vital-sign records. Blood pressure measurements during the predefined observation window were extracted to calculate BPV indices. Serum tau protein was measured from the first venous blood sample obtained at admission according to the protocol described in Section 2.2. Outcome status at the prespecified endpoint was obtained from the medical record or follow-up documentation and used for subsequent analyses.

Inclusion criteria were: (1) age ≥18 years; (2) meeting the diagnostic criteria for TBI (10); (3) complete pre-hospital data available.

Exclusion criteria were: (1) use of immunosuppressants within 2 weeks prior to admission; (2) pregnancy or lactation; (3) death before hospital arrival; (4) history of stroke, or concomitant neurological diseases such as meningitis, epilepsy, Alzheimer’s disease, or psychiatric disorders; (5) multiple severe injuries involving other body systems; (6) malignant tumors; (7) hematological diseases; (8) autoimmune diseases; (9) death within 24 h of admission. Informed consent was obtained from all patients’ families, and the study protocol was approved by the hospital’s Ethics Committee. We excluded patients who died within 24 h of admission to ensure that all enrolled patients had a complete 24-h blood pressure monitoring period for BPV calculation. Consequently, the study cohort consists of patients who survived the initial 24-h period. Patients who died between 24 h and the 28-day outcome assessment were included in the analysis and, based on their 28-day GOS score of 1 (death), were classified into the poor prognosis group. This choice was made to focus on patients for whom the predictive value of early (first 24 h) BPV and admission tau could be meaningfully assessed, though it introduces a potential survivor bias and limits the generalizability of our findings to the most severely injured patients with ultra-early mortality.

### 2.2 Methods

#### 2.2.1 BPV calculation

BPV was assessed using hourly systolic and diastolic blood pressure measurements collected during the first 24 h after hospital admission (0–24 h). An ambulatory blood pressure monitor (DMS-ABP, Shanghai Ouqi Electronic Technology Co., Ltd., China) was applied immediately upon admission and automatically recorded SBP and DBP at 1-h intervals. The BPV observation window was anchored to admission; the time from injury to admission was recorded separately and thus approximated the interval from injury to initiation of BP monitoring. BPV indices were computed from SBP/DBP values within the 0–24 h window only, including the coefficients of variation for SBP and DBP (CV-SBP and CV-DBP), calculated as CV = SD/mean for the corresponding series. To ensure reliability of variability estimates, cases were included in BPV analyses only if ≥18 hourly readings were available within the 24-h window; otherwise, the case was excluded from BPV computation.

#### 2.2.2 Serum tau protein level measurement

A 3 mL venous blood sample was collected from TBI patients at the time of admission. For reproducibility, “at admission” was operationally defined as the first venous blood draw obtained immediately after arrival to the emergency department/ward and prior to in-hospital definitive interventions whenever feasible; the interval from injury to blood sampling was approximated by the recorded “time from injury to admission” (hours). The sample was centrifuged to obtain the upper serum layer, which was then stored at −80 °C until analysis. Serum tau protein levels were detected using a commercial ELISA kit (Jianglai Biotechnology, Shanghai, China; Cat. No. JL46220) according to the manufacturer’s instructions. The detection limit of the assay was 0.156 ng/L.

#### 2.2.3 Data collection

Patient data were collected, including gender, age, cause of injury, disease severity, underlying diseases (coronary heart disease, diabetes mellitus, hypertension), time from injury to admission, craniotomy surgery, pupillary dilation, subarachnoid hemorrhage, midline shift ≥5 mm, cerebral contusion, white blood cell count, and C-reactive protein levels. However, we did not adjust for the time from injury to admission in our statistical models. Since tau protein kinetics are time-dependent, the lack of this adjustment may confound the observed association between tau levels and prognosis. Additionally, detailed time-varying ICU interventions (e.g., sedatives/analgesics, vasopressors, ventilator settings, hyperosmolar therapy, and ICP/CPP-directed protocols) were not systematically quantified and were therefore not included as covariates.

### 2.3 Prognosis grouping

Patients were divided into poor and good outcome groups based on their prognosis at 28 days after admission. Poor prognosis was defined as a Glasgow Outcome Scale (GOS) score of ≤ 3 (scores range from 1 to 5) (11).

### 2.4 Statistical analysis

Statistical analyses were performed using SPSS software (version 28.0). Categorical variables (e.g., sex, cause of injury, and underlying diseases) are presented as number (percentage) and were compared using the *χ*^2^ test. Normality of continuous variables was assessed using the Kolmogorov–Smirnov test. Normally distributed continuous variables (including age, serum tau protein, and white blood cell count) are reported as mean ± standard deviation and were compared using the independent-samples *t*-test; when an ordered trend across severity strata was evaluated, a trend *F*-test was applied. Non-normally distributed continuous variables (including BPV indices, time from injury to admission, and C-reactive protein) are reported as median (P_25_, P_75_) and were compared using the Mann–Whitney *U* test (for two-group comparisons) or the Jonckheere–Terpstra test (for ordered trend across severity strata). When post-hoc pairwise comparisons were required after ordered testing, Bonferroni correction was applied. Ordinal variables (e.g., disease severity) were analyzed using the Wilcoxon rank-sum test (Mann–Whitney *U* test) for comparisons between two independent groups, consistent with the approach used for non-normally distributed continuous data. Correlations were assessed using Spearman’s rank correlation analysis. Multivariable logistic regression (SPSS “enter” method) and ROC curve analysis were employed to evaluate factors associated with 28-day prognosis and the assessment value of BPV combined with serum tau protein. The SPSS “enter” method indicates forced-entry (simultaneous) inclusion of prespecified covariates in a single step, rather than forward or stepwise selection. Odds ratios from logistic regression are expressed per one-unit increase in the predictor. For descriptive purposes and to facilitate clinical interpretation in univariate comparisons (Table 1), we also defined binary cut-offs: elevated CV-SBP and CV-DBP were defined as values greater than the 90th percentile of their distribution in the study cohort, and elevated tau protein was defined as a concentration above the median; this dichotomization strategy was informed by prior literature and clinical relevance. However, the primary logistic regression model and the combined ROC model rely exclusively on the continuous forms of these variables. To address potential confounding by tau sampling timing, we performed a sensitivity analysis by including time from injury to admission (hours) as a covariate in the multivariable logistic regression model; because this variable was non-normally distributed, it was entered as a continuous covariate (hours), and interpretation focused on the stability of the tau effect estimate. To benchmark the proposed biomarker/physiology model against standard bedside predictors, a baseline clinical model including admission GCS and pupillary response was considered. However, due to the inherent collinearity between GCS and the study’s primary grouping variable (disease severity), and to avoid model overfitting, a formal benchmarking analysis with a separate baseline model was not conducted in the current study. The contribution of GCS is acknowledged in the initial stratification (Table 2) and univariate analyses (Table 1). Formal quantification of the incremental prognostic value of adding BPV indices and serum tau beyond established clinical predictors should be addressed in future larger, multicenter cohorts with appropriate validation procedures. Formal CT severity scores such as Rotterdam or Marshall could not be calculated because the full set of required CT components was not systematically recorded in this cohort; therefore, we used the available CT features for benchmarking. The significance level (*α*) was set at 0.05. To avoid overinterpretation of model performance, we acknowledge that the combined model was developed and evaluated in the same cohort, which may lead to an optimistic AUC. No external validation dataset was available, and internal validation procedures (e.g., bootstrap or cross-validation) were not performed; thus, the reported model discrimination should be considered exploratory.

**Table 1:** Comparison of clinical data between TBI patients with different prognoses.

Variable	Poor prognosis group (*n* = 53)	Good prognosis group (*n* = 152)	*p*
Sex (male/female)	28/25	98/54	0.134
Age (years, $\bar{x}\pm s$ )	56.13 ± 10.23	53.82 ± 12.11	0.216
Cause of injury [*n* (%)]			
Violent assault	5 (9.43)	14 (9.21)	0.419
Fall from height	16 (30.19)	58 (38.16)	
Traffic accident	26 (49.06)	72 (47.37)	
Other	6 (11.32)	8 (5.26)	
Severity of injury [*n* (%)]			
Mild	5 (9.43)	49 (32.24)	<0.001
Moderate	15 (28.30)	53 (34.87)	
Severe	33 (62.26)	50 (32.89)	
Comorbidities [*n* (%)]			
Coronary heart disease	13 (24.53)	32 (21.05)	0.599
Diabetes mellitus	9 (16.98)	16 (10.53)	0.216
Hypertension	19(35.85)	47 (30.92)	0.508
Time from injury to admission [h, M (P_25_, P_75_)]	6.00 (5.00,8.00)	6.00 (4.00,7.00)	0.077
Craniotomy [*n* (%)]			
Yes	30 (56.60)	63 (41.45)	0.056
No	23 (43.40)	89 (58.55)	
Mydriasis [*n* (%)]	20 (37.74)	27 (17.76)	0.003
Subarachnoid hemorrhage [*n* (%)]	41 (77.36)	104 (68.42)	0.218
Midline shift ≥5 mm [*n* (%)]	36 (67.92)	61 (40.13)	<0.001
Cerebral contusion [*n* (%)]	33 (62.26)	82 (53.95)	0.293
White blood cell count (×10^9^/L, $\bar{x}\pm s$ )	11.86 ± 3.00	11.70 ± 3.25	0.760
C-reactive protein [mg/L, M (P_25_, P_75_)]	16.15 (10.49, 19.21)	14.11 (11.10, 16.08)	0.053
CV_SBP_ [M (P_25_, P_75_)]	0.15 (0.12, 0.18)	0.11 (0.09, 0.13)	<0.001
CV_DBP_ [M (P_25_, P_75_)]	0.12 (0.11, 0.14)	0.09 (0.08, 0.11)	<0.001
Tau protein (ng/L, $\bar{x}\pm s$ )	29.38 ± 4.32	22.82 ± 6.25	<0.001

*p*-values were obtained using *χ*^2^ tests for categorical variables, independent-samples *t*-tests for normally distributed continuous variables, and Mann–Whitney *U* tests for non-normally distributed or ordinal variables.

**Table 2:** Comparison of BPV and serum tau protein levels across different TBI severities.

Group	Cases	BPV [M (P_25_, P_75_)]		Tau protein (ng/L, $\bar{x}\pm s$ )
		CV_SBP_	CV_DBP_	
Mild TBI group	54	0.10 (0.08, 0.11)	0.07 (0.07, 0.08)	17.34 ± 3.55
Moderate TBI group	68	0.11 (0.10, 0.12)^a^	0.10 (0.09, 0.11)^a^	22.34 ± 4.58^a^
Severe TBI group	83	0.15 (0.12, 0.18)^ab^	0.12 (0.11, 0.14)^ab^	29.95 ± 4.12^ab^
*F*/*J-T*	—	9.853	10.555	323.105
*p* for trend	—	<0.001	<0.001	<0.001

Superscripts indicate *post-hoc* pairwise comparisons with Bonferroni adjustment (*α* = 0.017): ^a^*p* < 0.017 vs. mild TBI group; ^b^*p* < 0.017 vs. moderate TBI group.

## 3 Results

### 3.1 Study population and baseline characteristics

A total of 205 TBI patients admitted to our hospital between January 2022 and May 2025 were prospectively enrolled. The mean age was 54.42 ± 11.67 years (range: 23–87 years); there were 79 females and 126 males. Causes of injury included: violent assault (*n* = 19), fall from height (*n* = 74), traffic accident (*n* = 98), and others (*n* = 14). Based on the GCS score at admission (10), patients were divided into a mild TBI group (GCS 13–15, *n* = 54), a moderate TBI group (GCS 9–12, *n* = 68), and a severe TBI group (GCS 3–8, *n* = 83). The levels of CV ~ SBP~, CV ~ DBP~, and tau protein increased sequentially from the mild TBI group to the moderate and severe TBI groups (all *p* < 0.017), as shown in Table 2.

### 3.2 Correlation between BPV, serum tau protein levels, and TBI severity

**Figure 1:**
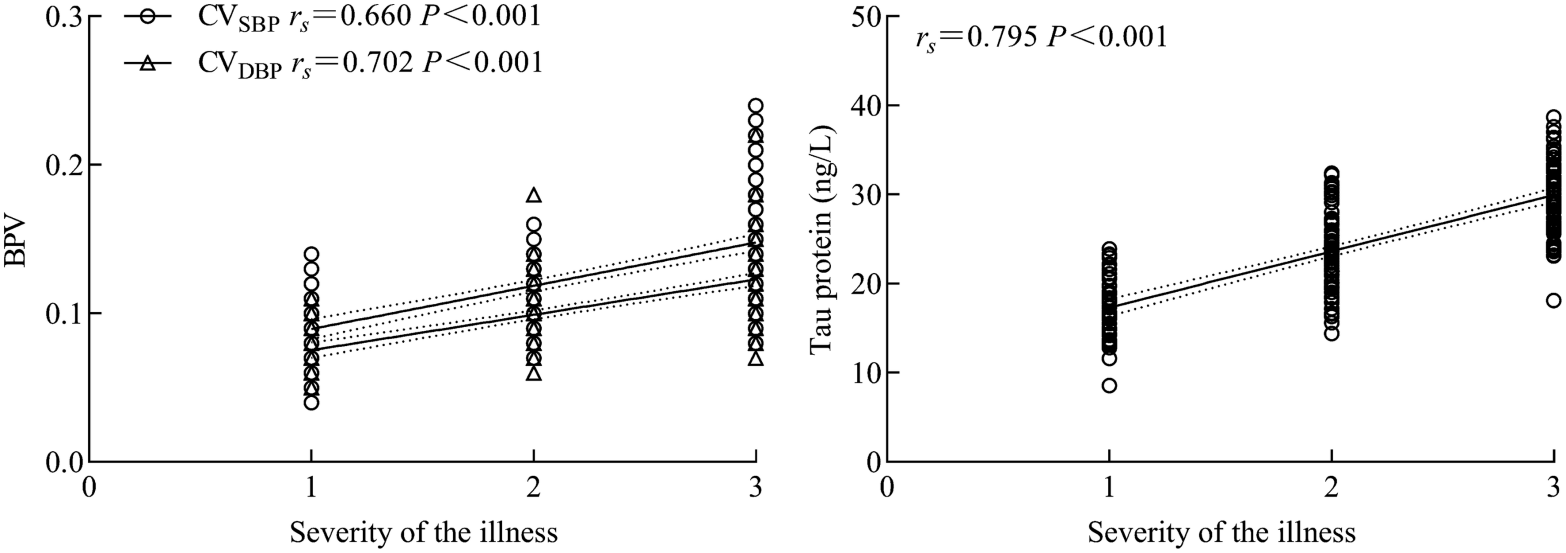
Blood pressure trajectories during the first 24 h after admission. **(A)** Systolic blood pressure (SBP) and **(B)** diastolic blood pressure (DBP) are presented in separate panels to improve visual discrimination between measures.

Spearman’s rank correlation analysis revealed that BPV and serum tau protein levels were positively correlated with the severity of TBI (*r*_s_ = 0.660, 0.702, 0.795, respectively; all *p* < 0.001), as illustrated in Figure 1.

### 3.3 Comparison of clinical data between TBI patients with different outcomes

The 28-day poor prognosis rate among the 205 TBI patients was 25.85% (53/205). All comparisons in Table 1 and subsequent regression/ROC analyses were based on this 28-day outcome definition (GOS ≤3). Compared to the good outcome group, the poor outcome group had a more severe initial condition, along with significantly higher proportions of pupillary dilation and midline shift ≥5 mm, as well as elevated CV ~ SBP~, CV ~ DBP~, and tau protein levels (all *p* < 0.05), as detailed in Table 1. The time from injury to admission did not differ significantly between the poor- and good-outcome groups (median 6.00 h in both groups, *p* = 0.077; Table 1). In sensitivity analyses additionally adjusting for time from injury to admission, the association between admission serum tau and 28-day poor outcome remained directionally consistent.

### 3.4 Multivariate analysis of poor prognosis in TBI patients

Using pupillary dilation (yes = 1/no = 0), midline shift ≥5 mm (yes = 1/no = 0), CV-SBP (entered as continuous raw value), CV-DBP (entered as continuous raw value), and tau protein (entered as continuous raw value) as independent variables (disease severity was excluded due to multicollinearity), and TBI patient prognosis (poor = 1/good = 0) as the dependent variable, a multivariable logistic regression model was established using the enter method. Multicollinearity was weak (all VIF <5). The results showed that pupillary dilation, midline shift ≥5 mm, elevated CV ~ SBP~, elevated CV ~ DBP~, and elevated tau protein were independent risk factors for poor prognosis in TBI patients (all *p* < 0.05), as shown in Table 3.

**Table 3:** Multivariate analysis of factors associated with poor prognosis in TBI patients.

Variable	*β*	SE	*p*	OR	95% CI
Mydriasis	1.105	0.547	0.043	3.020	1.034–8.819
Midline shift ≥5 mm	2.126	0.581	<0.001	8.381	2.682–26.190
CV-SBP (per 0.01 increase)	0.038^a^	0.080	<0.001	1.039^b^	1.025–1.053^b^
CV-DBP (per 0.01 increase)	0.040^a^	0.096	<0.001	1.041^b^	1.021–1.061^b^
Tau protein (per 1 ng/L increase)	0.307	0.059	<0.001	1.360	1.210–1.528
Constant	−20.160	3.111	<0.001	<0.001	—

OR, odds ratio; CI, confidence interval.

a The *β* coefficient for CV-SBP and CV-DBP has been adjusted to reflect a 0.01 unit increase for easier clinical interpretation.

b The OR and 95% CI for CV-SBP and CV-DBP have been recalculated accordingly to represent the effect of a 0.01 increase.

### 3.5 Assessment value of BPV combined with serum tau protein for poor prognosis in TBI patients

**Figure 2:**
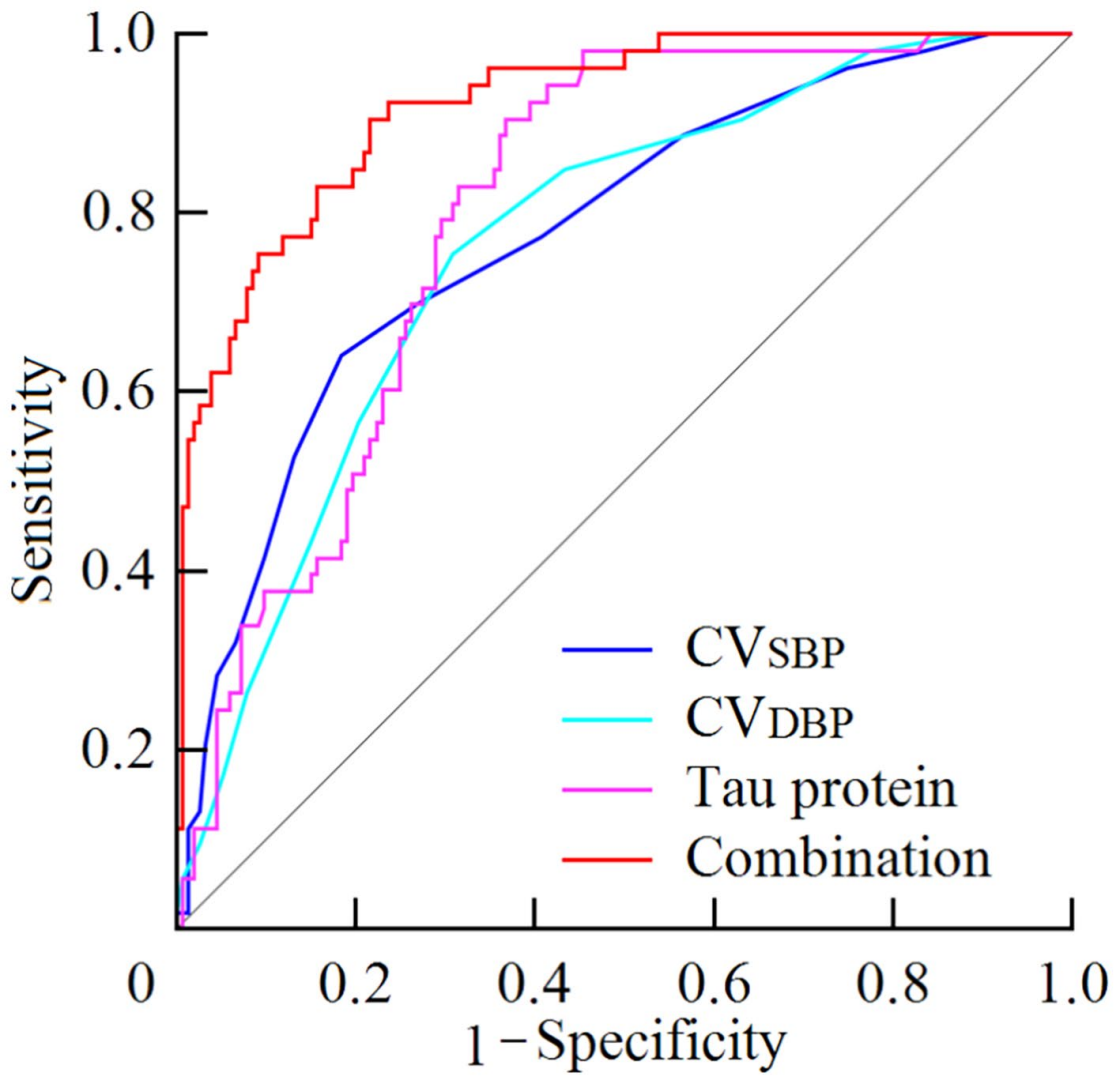
ROC curves of BPV, serum tau protein, and their combination for predicting poor prognosis in TBI patients.

The areas under the ROC curve (AUC) for CV ~ SBP~, CV ~ DBP~, serum tau protein level, and their combination in predicting poor prognosis were 0.778, 0.765, 0.799, and 0.921, respectively. The sensitivities were 64.15, 75.47, 90.57, and 84.91%, and the specificities were 81.58, 69.08, 63.16, and 80.26%, respectively. The Youden indices are shown in Table 4. According to the Delong test, the combined assessment value was superior to CV ~ SBP~, CV ~ DBP~, or serum tau protein level alone (Z = 4.437, 4.585, 4.391, respectively; all *p* < 0.001) (see Table 4 and Figure 2).

**Table 4:** Predictive value of BPV combined with serum tau protein for poor prognosis in TBI patients.

Indicator	Area under the curve	95% CI	*p*	Cut-off	Sensitivity (%)	Specificity (%)	Youden index
CV_SBP_	0.778	0.715–0.833	<0.001	0.13	64.15	81.58	0.4573
CV_DBP_	0.765	0.700–0.821	<0.001	0.11	75.47	69.08	0.4455
Tau protein	0.799	0.738–0.852	<0.001	25.01 ng/L	90.57	63.16	0.5372
Combination	0.921	0.875–0.954	<0.001	0.19^a^	84.91	80.26	0.6517

a Represents the fitted probability from the Logistic regression model using continuous predictors: Logit(*p*) = −16.369 + 3.32 × CV ~ SBP~ + 3.76 × CV ~ DBP~ + 0.258 × tau protein. Note that the coefficients for CV-SBP and CV-DBP in this equation correspond to a 1.0 unit increase. For a 0.01 unit increase, the coefficients would be 0.0332 and 0.0376, respectively.

## 4 Discussion

This prospective cohort study systematically investigated the individual and combined value of BPV and serum tau protein in assessing the prognosis of traumatic brain injury (TBI). Our primary findings are as follows: First, both BPV indices (CV ~ SBP~ and CV ~ DBP~) and serum tau protein levels significantly increased with the severity of TBI and showed strong positive correlations with the degree of injury. Second, in multivariate analysis, both were identified as independent risk factors for poor 28-day outcome in TBI patients. Third, the predictive model combining BPV and serum tau demonstrated higher discrimination (AUC = 0.921) than any single indicator in this cohort; however, given that model development and evaluation were performed in the same sample without internal resampling or external validation, these performance estimates should be considered exploratory and require confirmation in independent cohorts. While the AUC of 0.921 is promising, it is important to acknowledge that this value may reflect overfitting due to the relatively small sample size and the number of predictors in the model. Future studies should include internal validation procedures, such as cross-validation or bootstrap resampling, and perform calibration to assess the model’s generalizability and robustness. Additionally, decision curve analysis could be employed to evaluate the clinical utility of this predictive model. This study use the term “combined value” to describe the incremental predictive benefit of BPV and serum tau protein when used together, rather than implying a mechanistic interaction.

Despite ongoing improvements in standardized TBI management strategies based on guidelines, such as control of intracranial pressure, maintenance of cerebral perfusion pressure, surgical evacuation of hematomas, and comprehensive supportive care (12), mortality and disability rates, particularly for moderate and severe TBI patients, remain unacceptably high (13). However, our study excluded patients who died within 24 h of admission and those with multiple severe injuries, which may lead to a selection bias towards a more homogenous cohort of surviving patients with less severe comorbidities. This selection bias could inflate the predictive performance of BPV and tau protein, as these indicators may perform differently in more critically ill or comorbid populations. Future studies should aim to include these patients for a more comprehensive analysis. A significant number of survivors face long-term challenges from various neurological deficits, severely impacting their quality of life, family caregiving burden, and ability to reintegrate into society (14). This dire prognostic outlook underscores the extreme complexity of TBI’s pathophysiological mechanisms, which involve a cascade of interrelated processes including hemodynamic instability, inflammatory storms, oxidative stress, axonal injury, and even neurodegenerative changes. Therefore, clinical practice urgently demands tools that go beyond the limitations of the traditional GCS and conventional imaging to provide earlier, more objective, and accurate assessment of these core pathological processes, enabling effective prediction of individual patient outcomes. The results of this study suggest that combining BPV, reflecting macro-level physiological dysfunction, with serum tau protein, indicating micro-level neuronal structural damage, represents a significant step towards this goal.

Our study supports that BPV is a strong early prognostic marker associated with both TBI severity and outcome. This finding aligns with research on BPV in cerebrovascular diseases. Substantial evidence indicates that higher BPV is independently associated with poor functional outcomes and mortality in patients with acute ischemic stroke and intracerebral hemorrhage (16, 17). In the field of TBI, our study helps fill a gap in prospective evidence. While the retrospective study by Yuan et al. (18) linked CV ~ SBP~ to intraparenchymal hematoma progression, our prospective design extends the observed association of BPV to overall disease severity and functional outcome.

The mechanisms by which BPV may influence TBI outcome are likely multi-layered and interconnected. One plausible explanation is impaired cerebral autoregulation (CA) after TBI (7, 19). Under normal conditions, CA helps maintain stable cerebral blood flow (CBF) across a range of blood pressures by adjusting cerebral resistance vessels. Following TBI, CA is often disrupted, rendering CBF more dependent on systemic blood pressure (20). In this context, greater BP fluctuations captured by BPV may contribute to oscillations in cerebral perfusion pressure, with intermittent hypoperfusion (ischemia) and hyperperfusion (vasogenic edema and blood–brain barrier disruption) (8, 20). Consistent with this, our logistic regression results indicate that effects should be interpreted on the odds scale; based on the reported ORs per 1.0 unit increase in CV, a 0.01 increase in CV-SBP and CV-DBP corresponds to an estimated 0.38 and 0.40% increase in the odds of poor outcome, respectively [OR_(0.01) = 1.00380 and 1.00400]. BP fluctuations may also increase shear stress on vulnerable microvascular endothelium, compromising blood–brain barrier integrity and facilitating secondary neuroinflammation (10, 21). In addition, BPV has been linked to heightened sympathetic activity, which may further amplify inflammation and oxidative stress (22). Overall, BPV should be viewed as an early hemodynamic marker that may reflect impaired cerebrovascular regulation and systemic stress responses after TBI, rather than a proven causal driver. Importantly, early BP patterns in neurocritical care are influenced by management (e.g., sedation/analgesia, mechanical ventilation, vasoactive agents, hyperosmolar therapy, ICP/CPP-targeted strategies, and emergent surgery). Because these time-varying treatment factors were not fully quantified and adjusted for, the observed BPV-outcome association may partly reflect treatment intensity or underlying instability. Similarly, tau exhibits time-dependent kinetics after TBI; although measured at admission and examined with timing considerations, residual confounding related to pre-hospital course and sampling timing cannot be excluded. Future multicenter studies incorporating granular treatment/exposure variables and time-resolved modeling are needed to better address confounding.

Complementing BPV as a functional indicator, serum tau protein provides molecular evidence of neuronal injury. Our study showed that serum tau protein had the strongest correlation with TBI severity (*r ~ *s* ~ * = 0.795) and that in the continuous multivariable model, each 1 ng/L increase in serum tau protein was associated with a 36.0% increase in the odds of poor outcome (OR 1.360, 95% CI 1.210–1.528), highlighting its central role in assessing the severity of neuronal damage.

Tau protein is intrinsically an axonal microtubule-associated protein, essential for maintaining neuronal cytoskeletal stability and intracellular transport (23). During TBI, the immense mechanical forces cause axons to stretch, twist, and even rupture, resulting in diffuse axonal injury (DAI). This process directly leads to the release of intracellular tau protein into the extracellular space, eventually reaching the cerebrospinal fluid and bloodstream (11, 24). Consequently, serum tau protein levels can be regarded as a quantitative readout of the degree of axonal injury. The observed significant increase in its levels with worsening injury severity in our study is a direct reflection of the more widespread and severe DAI in severe TBI.

More importantly, the release of tau protein is not merely a passive consequence; it actively participates in the process of secondary injury. On one hand, the loss of functional tau protein from axons leads to the disintegration and destabilization of the microtubule skeleton, resulting in impaired axonal transport. This prevents neurons from receiving necessary nutrients and clearing metabolic waste, ultimately leading to neuronal dysfunction and apoptosis (25). On the other hand, extracellular tau protein, particularly in its aberrantly phosphorylated forms, itself exhibits “prion-like” neurotoxicity. It can be taken up by neighboring neurons and microglia, triggering and exacerbating neuroinflammation and oxidative stress (26, 27). For instance, phosphorylated tau can activate microglia, promoting the release of pro-inflammatory cytokines such as tumor necrosis factor-alpha (TNF-α) and interleukin-1β (IL-1β), forming a positive feedback loop of neuroinflammation (28). Furthermore, evidence suggests that tau pathology may be linked to long-term cognitive impairment and increased risk of Alzheimer’s disease following TBI (29). Thus, serum tau protein levels not only reflect the severity of the initial insult but also indicate the intensity of an ongoing, tau-driven secondary neurodegenerative process.

The most innovative finding of this study is the demonstration that the combined application of BPV and serum tau protein has a “1 + 1 > 2” predictive effect. From a pathophysiological perspective, this synergistic effect has a solid theoretical basis. We propose a potential “vicious cycle” model: the initial TBI causes axonal injury, releasing tau protein, and damages brainstem and higher cortical functions, leading to autonomic nervous system dysfunction and CA impairment, manifesting as increased BPV. The elevated BPV, in turn, exacerbates cerebral ischemia, BBB disruption, and neuroinflammation, further aggravating axonal and neuronal injury, leading to more tau protein release into the blood (10, 20, 30). This cycle continuously self-reinforces, collectively driving the patient towards a poor outcome.

At the clinical prediction level, the ROC analysis results perfectly validate this model. Individually, BPV (especially CV ~ SBP~) performed better in terms of specificity, suggesting its ability to better identify patients at high risk due to extreme hemodynamic instability. Serum tau protein, conversely, exhibited very high sensitivity, meaning it rarely misses patients with severe structural axonal injury. However, each used alone has its limitations: a patient with high BPV but minimal axonal damage may not have the worst prognosis, and conversely, a patient with high tau protein but stable hemodynamics might still have a chance for recovery. The combination of the two just right achieves complementary advantages between functional disturbance and structural damage, macro-physiology and micro-molecules, thereby enabling a more comprehensive and accurate identification of truly high-risk patients who are simultaneously subjected to both insults. This makes the combined model highly promising for guiding clinical decision-making. For instance, patients identified as very high-risk by the combined model might warrant more aggressive blood pressure stabilization therapy, earlier neuroprotective interventions, or closer multimodal monitoring.

Our study also reaffirms the established role of pupillary dilation and midline shift ≥5 mm as independent risk factors for poor outcome in TBI, consistent with numerous authoritative reports (31–33). Pupillary changes, especially loss of light reflex, are often critical signs of sharply increased intracranial pressure, direct brainstem compression, or ischemia, indicating extremely critical condition and impending irreversible brain damage. Midline shift ≥5 mm is an objective imaging marker of significant mass effect, suggesting severe hemispheric compression prone to herniation, compromised cerebral blood flow, and ischemic injury to deep structures. These indicators, due to their easy accessibility and strong predictive power, remain indispensable components of the clinical assessment system.

However, the core finding and greatest value of this study lie in our ROC curve analysis confirming that the predictive model combining the three indicators—BPV (CV ~ SBP~, CV ~ DBP~) and serum tau protein—for assessing prognosis was significantly more effective than any single indicator. This result has profound clinical implications. It indicates that BPV and serum tau protein provide complementary prognostic information from two distinct yet crucial dimensions: “function” and “structure.” BPV reveals the state of decompensated cardiovascular regulatory function and the risk of secondary injury, while tau protein quantifies the severity of primary axonal injury. A patient might have relatively stable blood pressure but severe axonal injury (high tau), or mild axonal injury but extremely unstable blood pressure (high BPV); assessment using either alone could miss the true risk. The combined model comprehensively captures these two different high-risk phenotypes, enabling a more holistic and precise identification of poor outcome risk. This provides clinicians with a powerful tool to earlier identify patients with the highest potential risk, potentially guiding more aggressive blood pressure stabilization strategies, earlier neuroprotective interventions, or closer multimodal monitoring, ultimately advancing truly individualized precision medicine.

Several limitations should be acknowledged. This was a single-center cohort, which may limit generalizability. The multivariable and combined prediction model was developed and evaluated in the same dataset, and performance metrics (including the AUC) may therefore be optimistic; internal validation (e.g., bootstrap resampling or cross-validation) and calibration assessment were not performed. In addition, we did not conduct hierarchical benchmarking against a standard clinical prognostic model incorporating admission neurological severity and established CT severity measures, nor did we formally quantify incremental predictive value from adding BPV indices and serum tau using nested-model comparisons (e.g., likelihood ratio testing) or comparative ROC analyses; these analyses should be addressed in future studies with complete severity scoring and validation. Moreover, we were unable to perform a dedicated sensitivity model explicitly adjusting for admission GCS in the current revision, and thus the independence of BPV indices and serum tau beyond GCS warrants confirmation in larger cohorts. Residual confounding and selection bias cannot be fully excluded despite prospective enrollment, because treatment strategies (e.g., blood pressure management, surgery, sedation) and other unmeasured neurocritical care interventions during the first 24 h may influence BPV and outcomes. Accordingly, the observed relationships should be interpreted as associations rather than causal effects, and the proposed model should be considered hypothesis-generating until validated and calibrated in independent, multicenter cohorts before clinical translation.

## 5 Conclusion

This study suggests that early BPV indices and serum tau protein levels are associated with TBI severity and 28-day outcome, and may serve as complementary early prognostic markers reflecting hemodynamic instability and axonal injury, respectively. Several limitations should be acknowledged. Excluding patients who died within 24 h and those with multiple severe injuries may have introduced selection bias and reduced the representativeness of the cohort, potentially limiting applicability to broader TBI populations. In addition, model performance was evaluated in the same cohort in which it was developed, and thus may be optimistic; internal validation (e.g., bootstrap resampling or cross-validation) and calibration assessment were not performed. Therefore, the proposed BPV-tau model should be considered exploratory, and future studies should confirm its incremental prognostic value beyond established clinical predictors and imaging features, and validate and calibrate the model in larger, multicenter prospective cohorts before clinical implementation. Further mechanistic studies are also warranted to elucidate the pathophysiological links between BPV and tau dynamics after TBI.

## Data Availability

The original contributions presented in the study are included in the article/supplementary material, further inquiries can be directed to the corresponding author.
